# Sudden sensorineural hearing loss: audiological profile during the COVID-19 pandemic

**DOI:** 10.3389/fneur.2024.1415068

**Published:** 2024-09-04

**Authors:** Kelly Abdo Peron, Marina Cançado Passarelli Scott, Tracy Lima Tavares Soeiro, Jônatas Bussador do Amaral, Sujana S. Chandrasekhar, Norma de Oliveira Penido

**Affiliations:** ^1^Department of Otolaryngology Head and Neck Surgery, Universidade Federal de São Paulo/Escola Paulista de Medicina, São Paulo, Brazil; ^2^ENT and Allergy Associates, LLP, New York, NY, United States

**Keywords:** sudden sensorineural hearing loss, sudden deafness, COVID-19, SARS-CoV-2, neurotology, audiology, audiological profile

## Abstract

**Introduction:**

Sudden sensorineural hearing loss (SSNHL) is an otological emergency that requires prompt recognition and intervention to prevent devastating impacts on people’s lives. During the COVID-19 pandemic, sensory deprivations have been reported in patients positive for SARS-CoV-2 virus, including deleterious effects on the auditory pathway. This study aims to describe the audiological profile of individuals with SSNHL during the COVID-19 pandemic and to correlate hearing recovery in subgroups of individuals with or without COVID-19.

**Methods:**

Prospective cohort including patients diagnosed with SSNHL evaluated in a tertiary care center between March 2020 and September 2022. Hearing loss was confirmed with pure-tone and speech audiometry, with Speech Recognition Threshold (SRT) and word recognition score (WRS). Audiometric testing was performed at the moment of diagnosis, then 7, 30 and 120 days after diagnosis. The average degree of hearing loss was assessed by calculating the 4-frequency pure tone average (4fPTA). The investigation of COVID-19 included RT-PCR technique for the SARS-CoV-2 virus and collection of information regarding disease severity. A statistical analysis was performed using an analysis of covariance (ANCOVA) model to compare the 4fPTA between the four groups (with and without a history of COVID-19, unilateral and bilateral cases) at the end of the follow-up period.

**Results:**

Fifty-two patients with SSNHL were assessed, 40 (76.9%) with unilateral and 12 (23.1%) with bilateral hearing loss, totaling 64 ears included. Of those, 15 (28.8%) patients tested positive for SARS-CoV-2 and were symptomatic for COVID-19. Of all unilateral cases, 22.5% were seropositive and showed symptoms of COVID-19, a number that increased to 50% for bilateral cases. Comparing the COVID-19 positive groups, individuals with unilateral SSNHL went from 40 dB as their average 4fPTA at onset to 20 dB as their average 4fPTA after 120 days, whereas those with bilateral SSNHL went from an initial average of 60 dB to a final average of 66 dB. Although the 4fPTA value of individuals with unilateral SSNHL improved in 7 days, the mean values showed no significant difference between positive and negative groups. There was a higher incidence of bilateral simultaneous SSNHL in patients who had not been vaccinated against COVID-19 and who presented with symptoms of severe COVID-19.

**Conclusion:**

Infection with SARS-CoV-2 resulted in more severe SSNHL, in bilateral SSNHL, and in poorer recovery from SSNHL in bilateral cases. Bilateral SSNHL was seen more frequently in patients who had not received vaccination against COVID-19.

## Highlights

- This study provides novel neurotology information focused on the occurrence and clinical profile of vestibulocochlear impairments in patients with Sudden Sensorineural hearing loss (SSNHL) prior to and during the COVID-19 pandemic.
- The recent data shows significant differences in hearing outcomes related to SSNHL when compared to the literature before the COVID-19 pandemic.
- By contrasting patients who tested positive and negative for SARS-CoV-2 with SSNHL in this study, we were able to pinpoint differences in the severity of hearing loss, number of affected ears and recovery of hearing thresholds.

## Introduction

Sudden sensorineural hearing loss (SSNHL) is typically reported by the patient as an abrupt sensation of hearing loss, confirmed by audiometric examination. It is defined as a sensorineural hearing loss with a reduction of 30 dB or more in at least three contiguous audiometric frequencies, occurring over a span of 72 hours (1). Unilateral involvement is much more common, with only 0.44 to 4.9% of cases occurring bilaterally (2, 3). Clinical reports of this type of condition were first described by Lake R. in 1908 (4) and then by De Kleyn in 1944 (5), in which various etiologies were put forward and, to this day, a large number of cases remain labeled ‘idiopathic’, with patients not receiving a precise etiological diagnosis.

Among the various proposed theories, there are vascular causes, immunological alterations, oxidative stress related hypothesis, as well as those based on viral infections, the latter of which was already suspected and described by Van Dishoeck in 1957 (6). The pathophysiological mechanisms involve viral incursion of the fluids and/or cochlear tissue or direct invasion of the cochlear nerve, via the hematogenous route, cerebrospinal fluid, or the middle ear (7). Another possibility is related to the reactivation of latent viruses in the inner ear or nerve tissue, with neurotropic pathogens resulting in cochleitis and/or viral neuritis. In addition, there may also be an indirect action, in which a systemic viral infection triggers an immune-mediated response or activates an oxidative stress response in the inner ear, thus triggering SSNHL (7). In 1997, using a combination of histological tests and molecular biology techniques to analyze the temporal bones of a patient with Ramsay Hunt Syndrome and SSNHL, Wackym identified the genomic DNA of the Var*icella Zoster Virus* (VZV) in the spiral and vestibular ganglion, as well as the geniculate ganglion of the affected side, suggesting that the latent virus may also be in the auditory pathways (8). Although not verified by audiometry, endolymphatic and perilymphatic viral precipitates were identified in temporal bone specimens of 10 patients with human immunodeficiency virus (HIV) disease during the acquired immunodeficiency syndrome (AIDS) crisis (9).

Although the symptoms of SSNHL have been exhaustively investigated over the last few decades, the search for its etiology remains challenging. Consequently, the lack of etiological definition in most cases makes it impossible to target the underlying disease, resulting in suboptimal hearing outcomes. Viruses are already known possible aggressors of the auditory pathway, but in the case of SARS-CoV-2, the exact mechanisms by which it affects the audio-vestibular system remain unclear (10) as pointed out by a systematic review. In the period of the SARS-CoV-2 pandemic, with the emergence of a new virus with neurotropic characteristics (11, 12), it became imperative to investigate COVID-19 and the audiological characteristics in patients affected by SSNHL.

The SARS-CoV-2 virus is responsible for the COVID-19, which has a wide range of clinical manifestations. Studies in the literature show that up to a quarter of the population can transmit the virus asymptomatically (13), while patients with risk factors might develop an exacerbated inflammatory response, with the possibility of fatal outcomes (14). In addition to the most common symptoms that accompany COVID-19 (such as those involving the upper and lower airways), sensory and neurological manifestations have also been reported, including hearing and vestibular alterations (15–17). In a subjective assessment of the degree of hearing discomfort, Freni et al. found that 40% of their sample experienced the onset or worsening of hearing loss during active SARS-CoV-2 infection (18).

Due to its neuropathogenicity, the SARS-CoV-2 virus has deleterious effects on the auditory pathway, from the peripheral up until the central systems (11, 12). Furthermore, an infection by SARS-CoV-2 may trigger endothelial damage that can lead to thrombotic events, a fact that is extremely important considering the terminal vascularization of the cochlea (19). In addition to acute conditions, neurotropic viruses can cause chronic neurological lesions and complications by mechanisms that have not yet been established (20). Similar mechanisms might also be associated with SARS-CoV-2, but due to its recent emergence and little follow-up time, it is not yet possible to establish a direct connection between viral activity and the subsequent immune responses generated. A recent study (21) has highlighted the potential effects of COVID-19 on the middle ear, suggesting a link between SARS-CoV-2 and otitis media with effusion onset as the virus can be detected in middle ear effusion post-Omicron infection, suggesting a risk of recurrence and emphasizing the need for otolaryngologist vigilance to ensure the proper treatment strategies are in place.

Regardless of the cause of the hearing loss, diagnostic confirmation from the onset of symptoms to the start of treatment should be carried out quickly, since there is a therapeutic window of approximately 2 weeks between the hearing event and the start of drug therapy for good recovery of hearing levels (22). After this period, the effectiveness of the treatment and the extent of hearing improvement are significantly reduced (22), and it may even be the only factor related to hearing recovery (23). Systemic corticosteroids are considered to have the best efficacy to date among the various therapeutical options (1, 24), but their efficacy has not been proven enough to date to be considered more than an option (not a recommendation) in the American Academy of Otolaryngology-Head and Neck Surgery (AAO-HNS) Clinical Practice Guidelines, updated in 2019 (1).

The current study aims to describe the audiological profile of individuals with sudden sensorineural hearing loss (SSNHL) during the COVID-19 pandemic and to correlate hearing recovery in subgroups of individuals infected and not infected by SARS-CoV-2.

## Methods

### Study design and inclusion criteria

A prospective cohort study was carried out involving patients at an outpatient clinic in a tertiary care center between March 2020 and September 2022. Patients of both sexes with a diagnosis of sudden sensorineural hearing loss were included, confirmed by means of pure-tone audiometry, speech audiometry and tympanometry, according to the criteria established by the AAO-HNS (1). In addition, it was essential that there were no cognitive alterations that made it impossible to perform the audiometric tests. Informed consent was obtained from every participant. In patients with a history of COVID-19 positivity, the infection had to precede SSNHL by a maximum of 6 months. The study was submitted to the University Research Ethics Committee and approval was obtained (#4.507.315).

Patients underwent structured history, demographic data collection and an otorhinolaryngological physical examination, carried out in outpatient consultations that took place weekly for the first month, then monthly until 6 months of follow-up. The investigation of COVID-19 positivity was carried out using the RT-PCR technique for the SARS-CoV-2 virus either during hospitalization or through tests performed at external clinics, and information regarding the severity of the condition was also collected during anamnesis. COVID-19 cases were classified as mild in individuals with non-specific symptoms, such as cough, sore throat, fever, chills, as well as clinical evolution without the need for hospitalization. Severe clinical involvement was considered in patients with Severe Acute Respiratory Syndrome (SARS) with complications, need for respiratory support and hospitalization, which was mainly in an intensive care unit (ICU).

### Audiometric assessment

Confirmation of the diagnosis was based on the AAO-HNS criteria (1), employing the strict definition of sudden sensorineural hearing loss, with a threshold reduction of 30 dB or more, in at least three contiguous audiometric frequencies, occurring over a 72-h time window. An Interacoustics AZ7 audiometer, calibrated annually, was used to test airway hearing thresholds at 250 Hz to 8,000 Hz, bone conduction at 500 Hz to 4,000 Hz, Speech Audiometry, Speech Recognition Threshold (SRT) and word recognition score (WRS). The masking used was Speech Noise and Narrow Band. Tympanometry analysis was also carried out.

Audiometric testing was performed at the time of the suspected diagnosis, followed by further tests at pre-established intervals of 7, 30 and 120 days. The Pure Tone Average (PTA) was calculated using air conduction thresholds of 500, 1,000, 2000 and 4,000 Hz. The 4-frequency pure tone average (4fPTA) value for bilateral cases was calculated by taking the arithmetic mean between the two affected ears. Hearing loss (HL) was measured according to the World Health Organization (WHO) classification (25): Normal hearing thresholds <20 dB, mild HL thresholds between 20 and 35 dB, moderate HL thresholds between 35 and 50 dB, moderately severe HL thresholds between 50 and 65 dB, severe HL thresholds between 65 and 80 dB, profound HL thresholds between 80 and 95 dB, and complete hearing loss thresholds >95 dB. The audiogram configuration was based on the Silman and Silverman (26) classification adapted from Carhart (27) and Lloyd and Kaplan (28). The symmetry or asymmetry criteria were based on the American Speech Language Hearing Association (ASHA) recommendations (29). We considered functional hearing for 4fPTA values to be less than 50 dB (1).

### Imaging and laboratory tests

Laboratory tests, using peripheral blood, and magnetic resonance imaging (MRI) were conducted according to an investigation protocol supported by the accepted literature (1).

### Treatment

All the patients included in the study were treated with oral corticosteroids, specifically prednisone 1 mg/kg/day as a single daily dose, with maximum dose of 60 mg/day, for 14 days, with tapering of the dose over 3 weeks. If the first week’s follow-up audiogram showed complete hearing recovery, the dose reduction began on day 7, not day 14. In patients with inadequate control of hypertension and/or diabetes mellitus, equivalent doses of deflazacort were used. Rescue therapy with intratympanic corticosteroids was not used in this study.

### Statistical analysis

The results obtained from the clinical assessment and audiometric tests were subjected to comparative statistical analysis. Quantitative variables were analyzed using means, medians, minimum and maximum values; categorical variables were evaluated as frequencies and percentages.

An analysis of variance model of the factors and the interaction between them was used to see if the mean initial 4fPTA values differed between the affected ears (Bilateral; Unilateral), between patients with and without a history of COVID-19 and between the combined effects of the two factors. Bonferroni correction was used for multiple comparisons.

Evolution in 4fPTA values between the four groups (with and without a history of COVID-19, unilateral and bilateral cases) over the 120 days (0–7–30-120 days), were tested using linear mixed-effects models with 1st order autoregressive covariance structure, adjusted by initial PTA measurements, focusing on analyzing the change in PTA throughout the follow-up period in relation to the initial value. Bonferroni correction was used.

The mean audiometric frequencies at the end of follow-up were compared between groups (patients with or without a history of COVID-19) using an analysis of covariance (ANCOVA) model. The dependent variable was the audiometric measurements obtained at the end of follow-up, the group as the independent variable and the measurements at baseline as the covariate. Comparisons of means between groups at baseline were made using Student’s t-test.

Statistical significance was set at *p* < 0.05. The analyses were carried out using the SAS v 9.4 program (SAS Institute, Inc., 1999).

## Results

### Study subjects

A total of 79 individuals diagnosed with sudden sensorineural hearing loss between March 2020 and September 2022 were evaluated. 27 individuals were excluded due to refusal to participate in the study or lack of adequate follow-up. The final sample of this study consisted of 52 volunteer patients, with 40 patients (76.9%) presenting with unilateral hearing loss and 12 patients (23.1%) with bilateral hearing loss. A total of 64 ears were included in this study.

Of the 12 bilateral cases, 11 had simultaneous involvement in both ears and, of these, 5 had symmetrical loss and 6 had asymmetrical loss. Only one patient presented with hearing loss at different times, with asymmetrical loss between the ears; that individual was considered to have sequential bilateral SSNHL. Among the 40 patients with unilateral SSNHL, the right ear was affected in 15 and the left in 25. 30 individuals were male (57.7%) and 22 were female (42.3%), with a ratio of 1.3 to 1. The average age of the study population was 44.71 ± 16.61 years (median 51 years), with only 3 (5.8%) individuals under the age of 18. 60% of the individuals reported chronic comorbidities, with a higher prevalence of systemic arterial hypertension (28.8%) and diabetes mellitus (17.3%). All of the patients complained of tinnitus.

### COVID-19 and follow-up

Fifteen (28,8%) patients had COVID-19 within the preceding 6 months. Of the unilateral cases, 22.5% were positive for COVID-19, while 50% of the bilateral cases had COVID-19, as shown in Table 1. 9 of the COVID-19 positive individuals had unilateral SSNHL while the remaining 6 had bilateral SSNHL.

**Table 1:** Demographic assessment of patients with sudden sensorineural loss collected from March 2020 to September 2022.

	Unilateral *n* (%)	Bilateral *n* (%)	Total *n* (%)
Patients with SSNHL	40 (76,9)	12 (23,1)	52 (100)
Sex			
Female	18 (45)	4 (33,3)	22 (42,3)
Male	22 (55)	8 (66,7)	30 (57,7)
Age			
Age per years^1^	46,85 ± 15,9	37,58 ± 17,61	44,71 ± 16,61
Median	51	44,5	51
Range	13–73	13–65	13–73
SARS-CoV-2 infection^2^			
Positive	9 (22,5)	6 (50)	15 (28,8)
Negative	31 (77,5)	6 (50)	37 (71,2)

^1Mean ± standard deviation. 2Previous history of SARS-CoV-2 infection within 6 months prior to SSNHL.^

Regarding the degree of the disease, 10 patients developed mild symptoms (9 unilateral and 1 bilateral case), while 5 developed severe symptoms (all bilateral cases), requiring prolonged hospitalization and a resultant delay in audiological testing. In particular, the patients considered to have severe COVID-19 had bilateral hearing impairment and all of them reported hearing loss after their sedation was suspended and the baseline neurological level was reestablished. These individuals required ICU admission and had extremely serious clinical complications, requiring orotracheal intubation (OTI) with sedation, hemodialysis and/or the use of ototoxic drugs. Among these, three patients also suffered cardiorespiratory arrest. Another unique feature of the patients with bilateral involvement and COVID-19 positivity, including the individuals with severe and mild evolution, is that they had not yet received COVID-19 vaccinations through the national immunization program.

When assessing study subjects in the other groups (COVID-19 negative bilateral, and COVID-19 negative and COVID-19 positive unilateral) we found mixed vaccination statuses, with vaccinated and unvaccinated cases in all subgroups, as well as different numbers of doses received at the time the SSNHL occurred. After introduction of the vaccination scheme, we found no cases of severe disease progression in the sample.

### Hearing assessment and recovery

Table 2 shows the data from the initial hearing assessment of the 64 ears studied, subdivided in relation to the affected ear(s), showing that the average degree of hearing loss assessed by calculating the 4fPTA of the two groups comprised the classification of moderately severe hearing loss (25). The configuration of the audiometric curve was classified as: descending in 45.3%, ascending in 20.3% and horizontal (or flat) in 17.2%. Of the total sample, five individuals (four unilateral cases and one bilateral) had predominant hearing loss in frequencies not used to calculate the tetratonal average. Of these cases, the patient with bilateral involvement had descending loss in both ears, two individuals with unilateral loss had marked descending loss and two others had an inverted U-shaped curve, whose values obtained by calculating the 4fPTA used in the study did not interfere with a change in functional classification and were therefore considered to be normal hearing.

**Table 2:** Initial hearing profile, by affected ear, of patients with sudden sensorineural loss (64 ears).

Ears affected	Unilateral 40 ears	Bilateral 24 ears (D/E)^#^	
Average initial 4fPTA (dB)	55.78 dB	60.89 dB (60.72 dB, 61.04 dB)	
Degree of hearing loss			
Normal	4	1	1
Mild	7	1	0
Moderate	6	2	3
Moderately severe	9	3	3
Severe	7	3	3
Profound	2	2	2
Complete	5	0	0
Curve configuration			
Descending			
Lightweight	5	0	0
Accentuated	6	3	1
On ramp	7	4	3
Horizontal	5	3	3
Ascending	10	1	2
U	2	1	2
Inverted U	5	0	1

Degree of hearing loss WHO 2020 (24).^#^D: right ear. E: left ear.

In patients who had had COVID-19, unilateral SSNHL occurred with a median of 68 days, while the bilateral cases occurred with a median of 41 days after infection. A wide variation in the number of days between the events was observed in both groups, as shown in Table 3.

**Table 3:** Hearing profile of patients with SSNHL and SARS-CoV-2 infection^1^ (15 individuals - 21 affected ears).

	Unilateral *n* (%)	Bilateral *n* (%)
Ears affected	9 (42,9)	12 (57,1)
Severity of COVID-19		
Mild Evolution	9 (42,9)	2 (9,5)
Severe Evolution	0	10 (47,6)
Average initial 4fPTA^2^	40.28 dB	60.10 dB (57.29 dB, 62.91 dB)^3^
Average final 4fPTA^2^	20 dB	65.93 dB (64.79 dB, 67.08 dB)^3^
Time (days) from COVID-19 to SSNHL^4^	78 ± 62,25	46 ± 27,9
Median	68	41
Interval of days	7–183	16–96
Time (days) from SSNHL to audiological examination^4^	16,56 ± 30,49	81,50 ± 62,64
Median	6	61
Interval of days	1–97	28–194

^1^Previous history of SARS-CoV-2 infection within 6 months prior to SSNHL.^2^Average initial and final 4fPTA were based on the first and last audiometry performed by each individual during follow-up.^3^Average 4fPTA of the two affected ears; Average 4fPTA per ear (right/left).^4^Mean ± standard deviation.

Table 4 describes the 37 individuals without SARS-CoV-2 infection, with 31 unilateral and 6 bilateral cases. When calculating the 4fPTA, the unilateral and bilateral cases had hearing loss with the same functional classification, both in the initial 4fPTA and at the end of follow-up. Outpatient follow-up and audiological documentation began for the unilateral and bilateral cases in an average of 13 and 4.8 days, respectively.

**Table 4:** Hearing profile of patients with SSNHL and absence of SARS-CoV-2 infection (37 individuals - 43 affected ears).

	Unilateral *n* (%)	Bilateral *n* (%)
Ears affected	31 ears (72.1)	12 ears (27.9)
Average initial 4fPTA^1^	60.28 dB	61.67 dB (64.16 dB, 59.16 dB)^2^
Average final 4fPTA^1^	43.83 dB	44.37 dB (47.08 dB, 41.66 dB)^2^
Time (days) from SSNHL to audiological examination^3^	13 ± 24,89	4,83 ± 1,94
Median	6	5
Interval of days	1–136	2–8

^1^Average initial and final 4fPTA were based on the first and last audiometry performed by each individual during follow-up.^2^Average 4fPTA of the two affected ears; Average 4fPTA per ear (right/left).^3^Mean ± standard deviation.

The analysis of variance presented in Table 5 shows that the mean value of initial 4fPTA did not differ between the affected ears, nor did it differ between patients with or without COVID-19.

**Table 5:** Analysis of variance of the mean values of initial 4fPTA per affected ear, associated or not with COVID-19.

Factors	*F*-statistics	*p*-value
Involvement of the ears		
Bilateral	1,39	0,2,438
Unilateral		
COVID-19		
Absence	1,44	0,2,361
Presence		
Ears * COVID-19	1,05	0,3,100

Table 6 compares the 4fPTA values between the unilateral and bilateral groups, in relation to whether they were positive for COVID-19 over the follow-up period.

**Table 6:** Comparison of 4fPTA between the unilateral and bilateral groups, in relation to positive and negative history for COVID-19, throughout the follow-up period.

	Group^*^	*p*- value	*p*- value^#^								
	Bilateral Negative (1)	Bilateral Positive (2)	Unilateral Negative (3)	Unilateral Positive (4)	Group X time	(1) x (2)	(1) x (3)	(1) x (4)	(2) x (3)	(2) x (4)	(3) x (4)
4fPTA^&^					0,013						
Initial	61,67 ± 6,19	60,10 ± 11,49	60,28 ± 4,73	40,28 ± 9,58						-	-
7 days	-8,55 ± 7,50	-1,01 ± 7,50	-6,95 ± 3,30^a^	−15,57 ± 6,28^a^		1,000	1,000	1,000	1,000	0,844	1,000
30 days	−15,74 ± 7,50^a^	9,68 ± 7,98	−17,39 ± 3,30^c^	−23,79 ± 6,49^c^		0,133	1,000	1,000	0,013	0,009	1,000
120 days	−16,83 ± 7,50^a^	7,84 ± 8,23	−15,90 ± 3,50^c^	−25,28 ± 6,59^c^		0,200	1,000	1,000	0,045	0,013	1,000

*Values expressed as mean ± standard error.^#^Values for comparison between groups of changes in follow-up time in relation to baseline were calculated using mixed-effects models with an autoregressive variance and covariance matrix. Bonferroni correction was used to adjust comparisons between groups.^&^Change in the 4fPTA in 7, 30 and 120 days in relation to the initial value.^a^*p* < 0.05 for comparison of the mean values at follow-up with the mean value at baseline within the group was calculated using a mixed-effects model with autoregressive variance and covariance matrix.^b^*p* < 0.01 for comparison of the mean values at follow-up with the mean value at baseline within the group was calculated using a mixed-effects model with autoregressive variance and covariance matrix.^c^*p* < 0.001 for comparison of the mean values at follow-up with the mean value at baseline within the group was calculated using a mixed-effects model with autoregressive variance and covariance matrix.

The mean 4fPTA values in patients with bilateral SSNHL and who were negative for COVID-19 showed significant hearing recovery throughout follow-up, except at 7 days, while patients with bilateral loss who were positive for SARS-CoV-2 showed no significant difference in relation to the initial value at any point in the analysis. This demonstrates that bilateral acoustic injury with positive history for COVID-19 showed the worst hearing recovery progression among the subgroups. Despite the different evolution, when assessing individualized hearing performance, we found no statistically significant difference when comparing these two groups.

In those with unilateral hearing impairment, patients with positive or negative results for the virus showed significant longitudinal changes compared to the initial 4fPTA values, without difference between the two groups.

**Figure 1:**
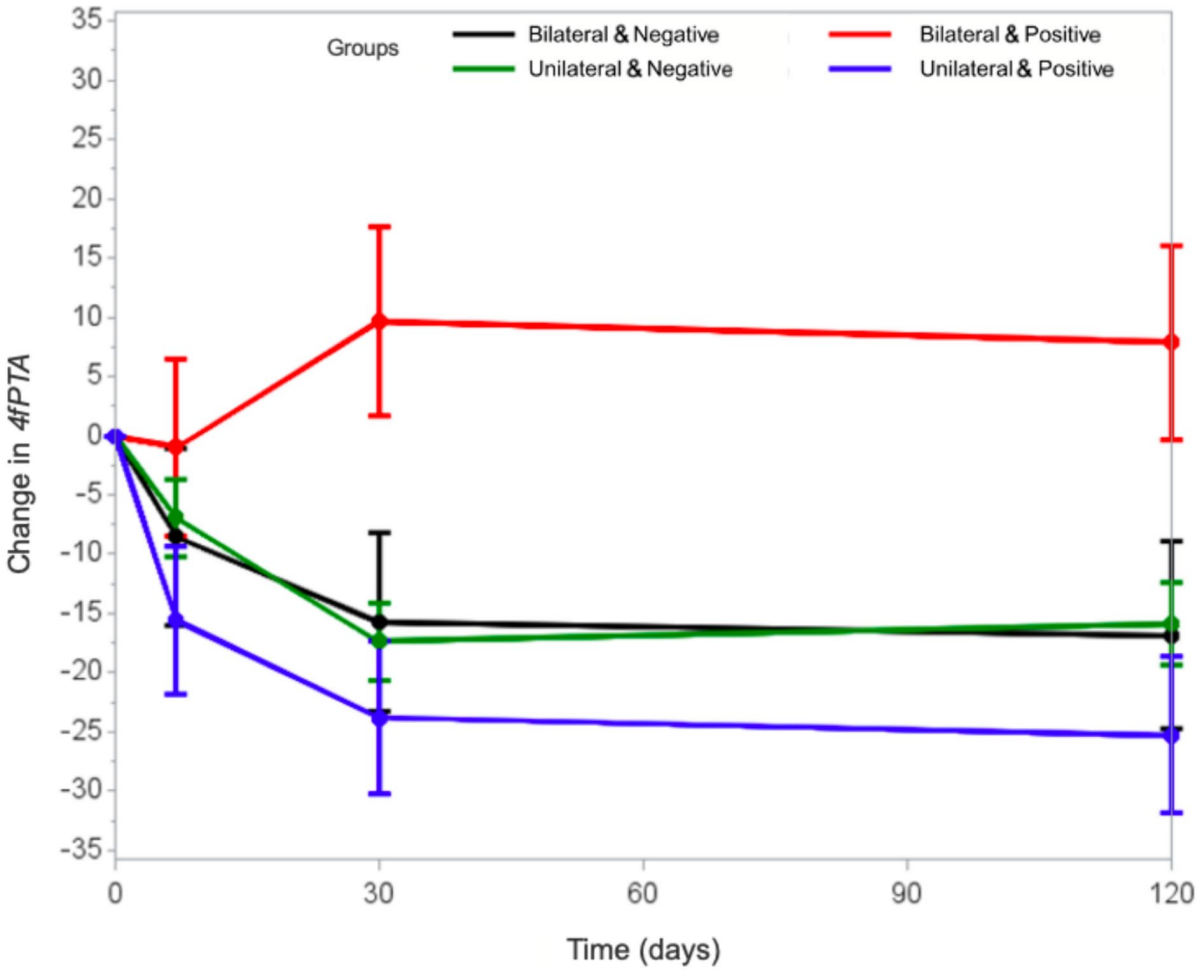
Comparison between the evolutionary curves of the 4fPTA in the unilateral and bilateral groups, in relation to the positive and negative history for COVID-19.

Figure 1 highlights the evolutionary discrepancy of the 4fPTA in individuals positive for SARS-CoV-2 who had bilateral involvement compared to the others. These patients demonstrated a tendency toward hearing deterioration, while in the other groups hearing improved. In some cases, the hearing improvement was not statistically significant, but the curves showed at least some degree of hearing recovery.

We compared the COVID-19 positive groups to each other. Those COVID-19 positive individuals with unilateral SSNHL had a significantly greater improvement in 4fPTA values at 30 and 120 days, compared to their hearing at presentation, and to the hearing of patients with COVID-19 and bilateral SSNHL. The 4fPTA value of individuals with unilateral SSNHL improved in 7 days, regardless of whether there was SARS-CoV-2 infection.

## Discussion

The patients in our study had a mean age of 44.7 years, similar to that reported in the literature (30–32) with a predominance of individuals over the age of 18. Only 3 individuals under the age of 18 were affected by the symptom of SSNHL, confirming the low incidence reported in the literature (33). Our pediatric patients had no clinical history related to ear infections, nor were they positive for COVID-19.

Tinnitus was observed in all individuals in our sample, which aligns with existing research indicating a high incidence of tinnitus associates SSNHL (1, 2, 34, 35).

In terms of hearing characteristics, we found 40 individuals with unilateral involvement and another 12 individuals with bilateral involvement. We found 6 ears with hearing classified as normal after a SSNHL episode, according to the WHO 2020 hearing classification (25). Because we used the 4fPTA value as the average of 4 frequencies (500, 1,000, 2000 and 4,000 Hz), descending losses with a predominance in the higher frequencies might not have been classified as a significant hearing loss. Therefore, the 4fPTA may not fully capture all auditory frequencies affected by hearing loss, potentially underestimating the severity and classification of the loss. While the 4fPTA might be consistent in classification, auditory perception and the impact of bilateral hearing loss vary among individuals. Both groups studied exhibited similar curve configurations, predominantly showing descending trends.

Our study found a notably high rate of bilateral SSNHL, with most cases being simultaneous. This contrasts with current literature and may be linked to the severity of COVID-19 in these patients (30, 36, 37). Additionally, nearly two-thirds of the bilateral cases had asymmetrical hearing loss, although symmetrical curve characteristics were more common in the literature (35). Despite the rarity of bilateral SSNHL before the COVID-19 pandemic, we found studies indicating that most SSNHL cases associated with COVID-19 were bilateral (38, 39).

In this regard, our data contradicts the literature, since papers in the period prior to the COVID-19 pandemic show bilateral involvement with prevalence below 8%, even though most studies do not specify whether they are simultaneous or sequential (37, 40, 41). In a longitudinal study with a long follow-up of up to 20 years, performed at the same institution as this study, bilateral involvement was reported in values close to 22%, although they usually occurred sequentially (42). The possibility of bilateral hearing loss occurring sequentially may underestimate the diagnosis of bilateral hearing loss, since the SSNHL events in each ear may happen months or years apart. The manifestation of SSNHL as unilateral or bilateral, simultaneous or sequential, depends on different pathophysiological conditions, and should be considered phenotypically as different entities (30, 36, 43).

We noticed that bilateral cases are more often in patients with systemic diseases (43), especially when presented simultaneously (30, 36), and this was also visible in our study.

In the bilateral cases, those negative for COVID-19 arrived at their first specialist appointment earlier. This shows that bilateral hearing impairment, particularly in those with worse hearing levels, has a greater impact on the individual’s quality of life, making them seek a specialist as early as possible. Similar figures have been described for simultaneous bilateral cases prior to the COVID-19 pandemic (40). The severity of COVID-19 had a great impact on the delayed diagnosis in bilateral positive cases, which is in line with other studies in the literature (3, 44). Of the positive cases, five had severe complications of COVID-19, with prolonged hospitalization and sedation, a fact that culminated in all patients identifying their own bilateral hearing loss only after their neurological state returned to baseline levels. These cases were subjected to multifactorial aggressors, including the pathophysiological process of the viral disease itself, such as recurrent hypoxia and exacerbated inflammatory status (45), as well as the adverse effects of the treatments employed to control the subsequent complications, such as exposure to ototoxic medications (46).

In our sample, all the individuals with severe course of COVID-19 were infected during a period that preceded the start of the vaccination program. It is important to point out that since the introduction of the vaccination program against COVID-19, we have not identified any cases with severe symptoms in our study population.

The behavior of the mean values of initial 4fPTA in the two types of involvement (unilateral and bilateral) did not differ between patients with or without COVID-19. Regardless, hearing recovery was different. The group of patients with COVID-19 positive and bilateral SSNHL did not show satisfactory hearing recovery because there was no improvement on speech discrimination test, nor in 4fPTA thresholds. However, in the COVID-19 negative and bilateral SSNHL group, there was at least some degree of hearing improvement, in both speech discrimination and 4fPTA thresholds. We believe that both the underlying comorbidities (that are known to influence hearing recovery) (47, 48) and the systemic inflammatory injury caused by SARS-CoV-2 are factors that may have influenced the hearing recovery of these bilateral SSNHL COVID-19 positive patients.

The vast majority of patients with unilateral SSNHL had their first hearing documentation within the therapeutic window period (44). Of the positive and negative cases, 88.9 and 83.9%, respectively, underwent the hearing test within 15 days of SSNHL, factors that contribute to a favorable prognosis (1, 44). As for the unilateral and positive individuals, they had primarily mild COVID-19 symptoms without the need for hospitalization.

The 4fPTA hearing threshold values of the unilateral patients showed significant improvement in both the positive and negative individuals in relation to the initial mean, with no difference between them. This behavior was similar over the 120-day follow-up period. Although 4fPTA recovery was not statistically different between SARS-CoV-2 positive and negative unilateral patients, the final mean 4fPTA values were different in terms of recovery amplitude.

We also noticed great discrepancy in the evolution of the subgroups, especially regarding hearing deterioration of the 4fPTA of the positive bilateral cases. The literature describes that the vast majority of individuals recover their hearing levels within this initial 30-day period, but isolated cases might still show hearing recovery after this initial interval (31, 49).

When we analyze each frequency individually, we find that the best tonal thresholds are preserved in the low-mid frequencies, which include most speech phonemes, while the worst threshold recovery occurs in the higher frequencies.

This study has some limitations. There was a restriction on the sample number and data collection of hearing tests from volunteer patients due to the difficulties inherent in the COVID-19 pandemic period, including patients’ clinical conditions. Additionally, COVID-19 is a recently studied disease, and although the literature lists many symptoms of acute SARS-CoV-2 infection (50), the definition of long COVID-19 is broad and has not yet been well established (42). Based on the definitions described so far, we used the history of infection by SARS-CoV-2 in the 6 months preceding SSNHL. With such a wide time range between infection and hearing loss, the probability of a true viral etiology may be lower. Although we did not incorporate the 6-month follow-up recommended by the AAO-HNS guidelines (1), we found results of clinical importance and statistical significance. We also demonstrated a tendency toward recovery and stability 30 days after the SSNHL hearing event in all subgroups, that was maintained up until the 120 days follow-up. The lack of worldwide standardization for correlating SSNHL findings made it difficult to compare the results obtained. The selection of frequencies for calculating the average 4fPTA also varies in the literature, with authors using 3, 4 or even the average of all audiometric frequencies. These variations make it difficult to compare the various studies that attempt to elucidate a symptom reported worldwide.

Despite these limitations, this study provides a broad assessment of the clinical profile of patients with SSNHL during the SARS-CoV-2 pandemic with a long-term follow-up window (30 to 120 days), that we feel corroborates the hypothesis of viral etiology. Although many important auditory aspects have been identified, future studies will be necessary to elucidate the real pathophysiological mechanism underlying the viral insult in the inner ear and other sites of the auditory pathway in SARS-CoV-2, as COVID-19 is a systemic disease and can affect hearing from the sensory cells in the inner ear to the central auditory nervous system.

## Conclusion

In this study, carried out during the COVID-19 pandemic, there was association between the onset of SSNHL and the SARS-CoV-2 infection, with higher incidence of bilateral SSNHL in unvaccinated patients presenting with severe COVID-19. An unfavorable hearing improvement was observed in bilateral positive cases, both in terms of speech discrimination threshold and 4fPTA values. However, the initial degree of sudden sensorineural hearing loss among individuals with unilateral and bilateral involvement did not differ between patients with or without COVID-19. Hearing stability was mostly reached 30 days after the SSNHL hearing event in all subgroups and was maintained until the 120 days of follow-up. Studies with a larger number of patients are needed to better elucidate these conditions.

## Data Availability

The original contributions presented in the study are included in the article/supplementary material, further inquiries can be directed to the corresponding author.
